# Invasive ventilation modes in children: a systematic review and meta-analysis

**DOI:** 10.1186/cc9969

**Published:** 2011-01-17

**Authors:** Anita Duyndam, Erwin Ista, Robert Jan Houmes, Bionda van Driel, Irwin Reiss, Dick Tibboel

**Affiliations:** 1 Intensive Care Unit, Erasmus MC - Sophia Children's Hospital, PO Box 2060, 3000 CB Rotterdam, The Netherlands

## Abstract

**Introduction:**

The purpose of the present study was to critically review the existing body of evidence on ventilation modes for infants and children up to the age of 18 years.

**Methods:**

The PubMed and EMBASE databases were searched using the search terms 'artificial respiration', 'instrumentation', 'device', 'devices', 'mode', and 'modes'. The review included only studies comparing two ventilation modes in a randomized controlled study and reporting one of the following outcome measures: length of ventilation (LOV), oxygenation, mortality, chronic lung disease and weaning. We quantitatively pooled the results of trials where suitable.

**Results:**

Five trials met the inclusion criteria. They addressed six different ventilation modes in 421 children: high-frequency oscillation (HFO), pressure control (PC), pressure support (PS), volume support (VS), volume diffusive respirator (VDR) and biphasic positive airway pressure. Overall there were no significant differences in LOV and mortality or survival rate associated with the different ventilation modes. Two trials compared HFO versus conventional ventilation. In the pooled analysis, the mortality rate did not differ between these modes (odds ratio = 0.83, 95% confidence interval = 0.30 to 1.91). High-frequency ventilation (HFO and VDR) was associated with a better oxygenation after 72 hours than was conventional ventilation. One study found a significantly higher PaO_2_/FiO_2 _ratio with the use of VDR versus PC ventilation in children with burns. Weaning was studied in 182 children assigned to either a PS protocol, a VS protocol or no protocol. Most children could be weaned within 2 days and the weaning time did not significantly differ between the groups.

**Conclusions:**

The literature provides scarce data for the best ventilation mode in critically ill children beyond the newborn period. There is no evidence, however, that high-frequency ventilation reduced mortality and LOV. Longer-term outcome measures such as pulmonary function, neurocognitive development, and cost-effectiveness should be considered in future studies.

## Introduction

Ventilator-induced lung injury in critically ill children suffering from acute respiratory failure should be counteracted by adapting ventilation management to the cause of respiratory failure [1]. Ideally, management should be based on proven effective strategies. In a multicenter study, bronchiolitis was the most frequent cause of respiratory failure in infants (43.6%); pneumonia the most frequent cause in older children (24.8%) [2]. Mortality in that study was rare (1.6%); the median duration of ventilation was 7 days. Randolph suggested that in pediatric clinical trials long-term morbidity would be a more sensitive indicator of the effects of clinical ventilation interventions than would mortality or duration of ventilation [1].

Pediatric intensive care units worldwide use a wide variety of ventilation modes: high-frequency oscillation (HFO), pressure control (PC), synchronized intermittent mandatory ventilation, pressure support (PS), pressure-regulated volume control and, more recently, neurally adjusted ventilator assist [3,4]. The ventilation mode is often not targeted specifically to the underlying disease but rather is determined by the intensive care physician's experience, local pediatric intensive care unit policy and protocols, or outcomes of studies in adults [1,2,5]. An unambiguous international guideline is still lacking [1,5].

The objective of the present article is to systematically review the randomized controlled trials (RCTs) comparing ventilation modes used in critically ill children (from term born up to 18 years of age) on the following outcome measures: length of ventilation, oxygenation, mortality, chronic lung disease and weaning. We aimed to determine whether there is sufficient evidence to decide on the better mode.

## Materials and methods

### Search and selection

A systematic search was performed in the PubMed and EMBASE databases in September 2010. MeSH terms and keywords searched for in the titles, abstracts and keywords areas were 'artificial respiration', 'instrumentation', 'device', 'devices', 'mode', and 'modes', combined with the Boolean operators AND, OR. (Additional file 1 provides the complete search strategy.) The search was limited to RCTs or quasi-experimental studies, with age limit >28 days until 18 years. Only articles comparing at least two ventilation modes were selected for review. Articles on non-invasive ventilation, studies in premature neonates (< 37 weeks) and articles in other languages than English or Dutch were excluded. No limits were imposed on the publication date.

Two authors (AD, EI) independently reviewed abstracts and full-text articles to identify eligible studies. Reference lists of retrieved studies were hand-searched for additional articles.

### Quality assessment

The study quality and level of evidence were assessed on criteria established by the Dutch Institute for Healthcare Improvement CBO in collaboration with the Dutch Cochrane library (see Additional file 2 and Table 1) [6]. The major criteria were as follows: Was assignment to the study group randomized? Were investigators blinded? Was it an intention-to-treat analysis? Were the study groups comparable? Was there appropriate report of outcome results for each group and the estimated effect size? Consensus between the authors on the interpretation of the extracted data was achieved.

**Table 1:** Level of evidence

Level	Description of evidence
1++	High-quality meta-analyses, systematic reviews of RCTs, or RCTs with a very low risk of bias
1+	Well-conducted meta-analyses, systematic reviews of RCTs, or RCTs with a low risk of bias
1-	Meta-analyses, systematic reviews of RCTs, or RCTs with a high risk of bias
2++	High-quality systematic reviews of case-control or cohort studies; or high-quality case-control or cohort studies with a very low risk of confounding, bias, or chance and a high probability that the relationship is causal
2+	Well-conducted case-control or cohort studies with a low risk of confounding, bias, or chance and a moderate probability that the relationship is causal
2-	Case-control or cohort studies with a high risk of confounding, bias or chance, and a significant probability that the relationship is not causal
3	Non-analytic studies; for example, case reports, case series
4	Expert opinion

RCT, randomized controlled trial.

### Data abstraction

Authors AD and EI each independently recorded patient characteristics (sample size, age, respiratory failure), details of the ventilation mode and the period over which outcome variables were measured. Outcome variables considered were the following: length of ventilation (LOV), oxygenation, chronic lung disease, mortality and weaning.

### Statistical methods

We quantitatively pooled the results of individual trials, where suitable. We expressed the treatment effect as an odds ratio (OR) for dichotomous outcomes and as a weighted mean difference (WMD) for continuous outcomes with 95% confidence intervals (CIs). The pooled OR was estimated with the Mantel-Haenszel method, which is generally the most robust model [7]. Differences were considered statistically significant if *P *< 0.05 or if the 95% confidence interval did not include the value 1. The analyses were performed with Microsoft^® ^Excel, Office 2007 for Windows.

## Results

### Search and selection

**Figure 1:**
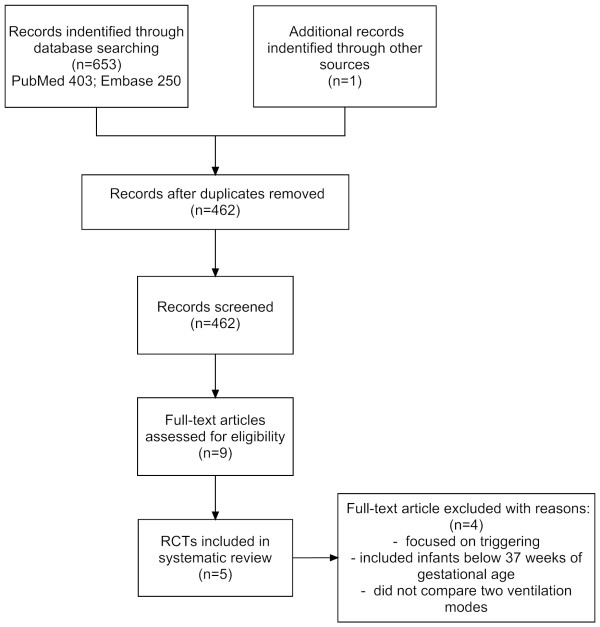
**Search results**. RCT, randomized controlled trial.

After filtering out duplicate studies, the titles and abstracts of 461 potentially relevant articles were screened (Figure 1). The reference lists yielded one other study that had been missed because the keywords were not in the title or abstract. Eventually, nine full-text articles were retrieved and assessed for eligibility. Four RCTs were excluded for any of the following reasons: focus on triggering instead of ventilation, inclusion of infants below 37 weeks of gestational age, or not comparing two ventilation modes [8-11]. The present review therefore includes five RCTs [12-16].

Tabulated details of these five RCTs are presented in Tables 2 and 3.

**Table 2:** Included randomized controlled trials - ventilation

Reference	Study population	Intervention/mode	Outcome measures				Level of evidence
				
			Mortality/survival	LOV (days)	Oxygenation	CLD	
Arnold and colleagues [12]	58 children (age: HFO 2.5 ± 2.5 vs. CV 3.1 ± 3.3 years) with diffuse alveolar disease and/or airleak syndrome	Multicenter study (five centers)	Number of survivors at 30 days - CV: 17 of 29 (59%); HFO: 19 of 29 (66%) (NS)	Total - CV: 22 ± 17; HFO: 20 ± 27	PaO_2_/PAO_2 _increase over time (72 hours) in HFO compared with CV (*P *< 0.001)	CV: *n *= 10 (59%); HFO: *n *= 4 (21%) (*P *= 0.039; OR = 5.4 95% CI = 1.2 to 23.2) (O_2 _at 30 days)	1+
		Comparison effectiveness of HFO (*n *= 29) with CV (*n *= 29) - crossover	Death (ranked) - CV: 40%, CV to HFO: 42%, HFO: 6%, HFO to CV: 82% (*P *≤0.001)	Survivors (at 30 days) - CV: 29 ± 18; HFO: 27 ± 31.	PaO_2_/PAO_2 _- HFO: 0.13 (0 hours) up to 0.26 (72 hours); CV: 0.13 (0 hours) up to 0.22 (72 hours)		
		Crossover: CV to HFO (*n *= 19), HFO to CV (*n *= 11)		Nonsurvivors (at 30 days) - CV: 11 ± 9; HFO: 8 ± 6 (NS)	After crossover - PaO_2_/PAO_2 _increase over time (72 hours) in CV to HFO group compared with HFO to CV group (*P *= 0.003)		
Dobyns and colleagues [14]	99 children (age 0 to 23 years) with AHRF, oxygenation index >15	Multicenter study (seven centers)	Trend of improved survival in HFO + iNO - CV: 22 of 38 (58%); CV + iNO: 20 of 35 (53%); HFO: 7 of 12 (58%); HFO + iNO: 10 of 14 (71%) (*P = *0.994)	CV: 22 ± 4; CV + iNO: 21 ± 3; HFO: 52 ± 28; HFO + iNO: 17 ± 4 (*P *= 0.098)	PaO_2_/FiO_2 _(PF) ratio - after 4 hours: HFO + iNO 136 ± 21 vs. CV 96 ± 6 (*P *= 0.2); after 12 hours: HFO + iNO 184 ± 45 vs. CV 107 ± 8 and CV + iNO 115 ± 9, HFO 136 ± 32 (*P *= 0.023); after 24 hours: treatment both HFO + iNO and HFO resulted in greater improvement in PF ratio than CV or CV + iNO (*P *= 0.005); after 72 hours: HFO 259 ± 60 vs. CV 148 ± 15 and CV + iNO 150 ± 19; HFO + iNO 213 ± 29 (*P *= 0.027)		1+
		Comparisons between patients treated with HFO + iNO (*n *= 14), HFO alone (*n *= 12), CV + iNO (*n *= 35), and CV alone (*n *= 38)					
Jaarsma and colleagues [13]	18 children (age 0 to 10 years) with respiratory failure for ventilation	Single-center study	ND	BIPAP: 9.8 ± 9.2; PS: 6.4 ± 5.8 (*P *= 0.27)	ND		1-
		Compare BIPAP (*n *= 11) with PS (*n *= 7), determining which mode is effective, safe and easy					
Carman and colleagues [16]	64 children (age 7.4 ± 0.7 years) with inhalation injury	Single-center study	VDR: 2/32 (6%); PC: 5/32 (16%) (NS)	VDR: 12 ± 2; PCV: 11 ± 2 (NS)	PF ratio - VDR: 563 ± 16; PC: 507 ± 13 (*P *< 0.05)		1-
		Compare VDR (*n *= 32) with PC (*n *= 32)					

Data presented as number/total (percentage) or mean ± standard deviation. AHRF, acute hypoxemic respiratory failure; BIPAP, biphasic positive airway pressure; CI, confidence interval; CLD, chronic lung disease; CV, conventional mechanical ventilation; HFO, high-frequency oscillation ventilation; iNO, inhaled nitric oxide; LOV, length of ventilation; ND, no data; NS, not significant; OR; odds ratio; VDR, volume diffusive respirator (high-frequency time-cycled pressure ventilator); PC, pressure-controlled ventilation; PS, pressure support ventilation.

**Table 3:** Included randomized controlled trials- weaning

Reference	Study population	Intervention/mode	Outcome measures			Level of evidence
				
			Duration of weaning (days)^a^	Extubation failure rate	Oxygenation	
Randolph and colleagues [15]	182 children (age 0 to 17 years) with weaning of ventilation support for more than 24 hours and who failed a test for extubation readiness on minimal PS	Multicenter study (10 centers) to evaluate weaning protocols comparing VS (continuous automated adjustment of PS by the ventilator) (*n *= 59) and PS (adjustment by clinicians) (*n *= 61) with standard care (no protocol) (*n *= 59)	PS: 1.6 (0.9 to 4.1); VS: 1.8 (1.0 to 3.2); no protocol: 2.0 (0.9 to 2.9) (*P *= 0.75)	PS (15%), VS (24%); no protocol (17%) (*P *= 0.44). Male children more frequently failed extubation (OR = 7.86 95% CI = 2.36 to 26.2; *P *< 0.001)	ND	1++

^a^Data presented as median (interquartile range). CI, confidence interval; ND, no data; OR, odds ratio; PS, pressure support; VS, volume support.

### Length of ventilation

The LOV served as the outcome measure in four studies (Table 2). First, Arnold and colleagues in a multicenter trial compared HFO and conventional ventilation (CV) in 58 children with either diffuse alveolar disease and/or air leak syndrome; 29 had been randomized to HFO, and 29 to CV [12]. During the first 72 hours of study, the mean airway pressure was significantly (*P *< 0.001) higher in the HFO group. The HFO strategy entailed aggressive increases in mean airway pressure to attain the ideal lung volume and to achieve an arterial oxygen saturation >90% with FiO_2 _< 0.6. The CV strategy entailed stepping up the end-expiratory pressure and inspiratory time to increase the mean airway pressure and to limit peak inspiratory pressure increases. Crossover to the alternate ventilator was required if the patient met defined criteria for treatment failure. LOV did not significantly differ between the CV and HFO groups (WMD = 2.0 days, 95% CI = -9.61 to 13.61).

Second, Dobyns and colleagues in a multicenter study compared HFO and CV in 99 children with acute hypoxemic respiratory failure [14]. Seventy-three children were treated with CV (38 without inhaled nitric oxide (iNO), 35 with iNO), and 26 with HFO (12 without iNO, 14 with iNO). Mechanical ventilation and FiO_2 _were adjusted to maintain SaO_2 _at 90% and pCO_2 _between 45 and 55 mmHg. Higher pCO_2 _values were tolerated as long as the arterial pH was 7.20. In the CV strategy, the positive end-expiratory pressure was increased incrementally to improve oxygenation while avoiding clinical and radiographic signs of lung hyperinflation. The peak airway pressure was maintained at <35 to 40 cmH_2_O by limiting the level of tidal volume and positive end-expiratory pressure. The initial HFO settings were: FiO_2 _of 1.0, 33% inspiratory time, frequency of 10 Hz, and mean airway pressure set at 2 to 4 cmH_2_O above that used on CV. The pressure amplitude was set to achieve perceptible chest wall motion and was adjusted if possible to optimize ventilation. In this study HFO did not lead to a significantly shorter LOV (Table 2). For the two ventilation groups without iNO, however, the LOV significantly differed between CV and HFO (WMD = -30.0 days, 95% CI = -45.89 to -14.11).

Third, Carman and colleagues compared the volume diffusive respirator (VDR) with PC ventilation in burned children with inhalation injury [16]. The VDR is a high-frequency, time-cycled pressure ventilator that can ventilate, oxygenate and promote secretion removal. SaO_2 _was maintained at or above 90%; PaCO_2 _was maintained at <55 mmHg. Thirty-two children with a mean ± standard deviation age of 5.5 ± 0.9 years were treated with VDR, and 32 children with a mean ± standard deviation age of 9.4 ± 1.0 years were treated with PC ventilation (*P *= 0.04 for mean age). The LOV was significantly different between the study groups (WMD = -1.0 days, 95% CI = -1.98 to -0.02).

Fourth, Jaarsma and colleagues randomized 18 children with respiratory failure to either biphasic positive airway pressure (*n *= 11) or pressure support ventilation (*n *= 7); their median age was 4 months (range 4 weeks to 10 years) [13]. Initial ventilator settings depended on age and the cause of respiratory failure, and were adjusted according to thoracic excursions and the measured tidal volume. Adjustments were made afterwards aiming at a pCO_2 _of 4 to 5 kPa and a pO_2 _of 8 to 11 kPa. The LOV did not significantly differ between biphasic positive airway pressure (9.8 ± 9.2 days) and PS (6.4 ± 5.8 days).

Pooled analysis of these trials resulted in a significantly shorter LOV after CV in comparison with HFO (WMD = -2.3 days, 95% CI = -3.63 to -1.04) (Table 4).

**Table 4:** Meta-analysis of trials comparing high-frequency ventilation with conventional ventilation: length of ventilation

Study	CV		HFOV		WMD (95% CI)	*Z *value (*P *value)
			
	Mean (SD)	*n*	Mean (SD)	*n*		
Arnold and colleagues [12]	22 (17)	29	20 (27)	29	2 (-9.61 to 13.61)	-0.338 (0.74)
Dobyns and colleagues [14]	22 (4)	38	52 (28)	12	-30 (-45.89 to -14.11)	3.699 (0.0002)
Subtotal		67		41	-11.51 (-15.14 to -7.88)	-6.221 (< 0.0001)
Carman and colleagues (VDR) [16]	11 (2)	32	12 (2)	32	-1 (-1.98 to -0.02)	-2.0 (0.046)
Overall		99		73	-2.34 (-3.63 to -1.04)	-3.542 (0.0004)

CI, confidence interval; CV, conventional ventilation; HFOV, High-frequency oscillation ventilation; SD, standard deviation; VDR, volume diffusive respirator (high-frequency time-cycled pressure ventilator); WMD, weight mean difference.

### Oxygenation

Three studies addressed the effects of different ventilation modes on oxygenation.

In the study by Dobyns and colleagues, the PaO_2_/FiO_2 _(PF) ratio improved most in the HFO mode with iNO after 4 hours (136 ± 21 mmHg vs. CV 96 ± 6 mmHg; *P *= 0.2) and after 12 hours (HFOV + iNO 184 ± 45 mmHg vs. CV 107 ± 8 mmHg and CV + iNO 115 ± 9 mmHg, *P *= 0.023; HFOV 136 ± 32 mmHg) [14]. After 24 hours, HFO treatment both with and without iNO provided better oxygenation than CV both with and without iNO (*P *< 0.05). After 72 hours, HFO treatment was associated with the best improvement in PF ratio (HFO 259 ± 60 mmHg vs. CV 148 ± 15 mmHg and CV + iNO 150 ± 19 mmHg, *P *= 0.027; HFOV + iNO 213 ± 9 mmHg). The two therapies did not differ in failure rate. Arnold and colleagues reported a significant (*P *= 0.001) relationship between time and a decreasing oxygenation index in the HFO group but not in the CV group [12]. After crossover (19 patients crossed over from CV to HFO and 11 patients crossed over from HFO to CV) this relationship was significant in both crossover groups (*P *= 0.03 crossover to CV; *P *= 0.02 crossover to HFO).

Carman and colleagues reported a significantly higher PF ratio in the VDR mode compared with PC (563 ± 15 mmHg vs. 507 ± 13 mmHg, *P *< 0.05) but did not specify the time point at which the best PF ratio was measured [16]. As the oxygenation parameters in these three studies were not uniform it was not possible to pool the data.

### Mortality and survival

Three studies focused on the outcome measure of mortality or survival.

None found a significant difference in mortality between patients treated with HFO and those treated with CV. Arnold and colleagues reported a mortality rate of 34% (10/29) for HFO versus 41% (12/29) for CV (OR = 0.75, 95% CI = 0.26 to 2.16) [12]. The mortality rate in patients not crossed over to CV from HFO or to HFO from CV, however, was significantly better (*P *= 0.003) than that in patients managed with CV only.

Dobyns and colleagues showed that the survival rate for patients treated with HFO in combination with iNO was higher than that for patients treated with HFO only or with CV (71% vs. 58% in CV, 53% in CV + iNO and 58% in HFO) [14]. These differences did not achieve statistical significance. These authors speculated that the improved lung recruitment by HFO enhances the effects of low-dose iNO on gas exchange. The mortality rate for HFO without iNO was 42% (5/12) versus 42% (16/38) for CV without iNO (OR = 0.98, 95% CI = 0.26 to 3.66) [14]. In the study by Carman and colleagues, five out of 32 (16%) patients in the PCV group died versus two out of 32 (6%) in the VDR group (OR = 0.36, 95% CI = 0.06 to 2.01) [16].

In the pooled analysis, the mortality rates in the HFO mode and in CV did not differ (OR = 0.70, 95% CI = 0.33 to 1.47) (Table 5).

**Table 5:** Meta-analysis of trials comparing high-frequency ventilation with conventional ventilation: mortality

Study	Conventional ventilation	High-frequency oscillation ventilation	Odds ratio (95% confidence interval)
Arnold and colleagues [12]	12/29	10/29	0.75 (0.26 to 2.16)
Dobyns and colleagues [14]	6/38	5/12	0.98 (0.26 to 3.66)
Subtotal Mantel-Haenszel	67	41	0.83 (0.30 to 1.91)
Carman and colleagues (VDR) [16]	5/32	2/32	
Overall Mantel-Haenszel	99	73	0.70 (0.33 to 1.47)

Data presented as number/total. VDR, volume diffusive respirator (high-frequency time-cycled pressure ventilator).

### Chronic lung disease

Chronic lung disease was examined only in the study by Arnold and colleagues [12]. The proportion of patients treated with HFO and requiring supplemental oxygen at 30 days was lower than that of patients managed with CV (*P *= 0.039; OR = 5.4, 95% CI = 1.2 to 23.2).

### Weaning

Randolph and colleagues randomized 182 children aged from 0 to 17 years to either a PS protocol (*n *= 62), a volume support (VS) protocol (*n *= 60) or a no ventilation weaning protocol in which weaning was at the discretion of the physician (*n *= 60) (Table 3) [15]. The VS and PS protocols dictated that FiO_2 _and positive end-expiratory pressure be adjusted to maintain SpO_2 _at 95% or higher. In the PS protocol, the amount of pressure support was adjusted to achieve an exhaled tidal volume goal of 5 to 7 ml/kg. In the VS protocol, the ventilator automatically adjusted the level of PS to achieve an exhaled tidal volume of 5 to 7 ml/kg.

Two outcome measures were assessed: weaning time and extubation failure (that is, any invasive or non-invasive ventilator support within 48 hours of extubation). The authors hypothesized that VS would result in a shorter weaning time as the inspiratory pressures automatically decrease with improvement of lung compliance. Most children could be weaned within 2 days and the weaning time did not significantly differ for the protocols used: PS, 1.6 days; VS, 1.8 days; and no protocol, 2.0 days. Extubation failure rates were not significantly different for PS (15%), VS (24%) and no protocol (17%).

### Quality of studies

These five studies compared six different ventilation modes in 421 children [12-14,16]. Two studies, based on an intention-to-treat analysis, met all CBO quality criteria [14,15]. Blinding was not possible in any of these studies, because ventilator displays cannot be masked. In four studies, patient characteristics and prognostic variables did not differ between the intervention groups. In the study by Carman and colleagues, the mean age differed significantly [16]. Only one study calculated the estimated effect sizes (relative risk of OR) for continuous outcome variables such as LOV, survival or weaning failure [15]. The study by Dobyns and colleagues [14] is of limited quality because it is a secondary analysis of data obtained from a previous multicenter, randomized trial on iNO treatment in pediatric acute hypoxemic respiratory failure [8]. The mode of ventilation was determined by the attending physician with the guidance of guidelines to maximize oxygenation. The patient was then randomized to treatment with or without iNO [14]. Levels of evidence for the different studies are presented in Tables 2 and 3.

## Discussion

The present review aimed at identifying the various ventilation modes used in children over the past three decades, searching for any data that would favor a particular mode for pediatric ventilation. The five RCTs included in this review varied in the investigated modes of ventilations, in outcomes and in patient groups.

High-frequency ventilators may use different ventilation modes. Two studies included in the present review concerned HFO ventilation [12,14]; a third concerned the VDR (high-frequency, time-cycled pressure ventilator) [16]. The evidence from these studies does not allow making a recommendation on the preferred type of high-frequency ventilator. Two RCTs compared HFO with CV on the outcomes oxygenation, LOV and mortality. Neither study found significant differences in mortality and LOV. Analysis of the pooled data, however, revealed a significantly lower LOV for the CV groups. A confounding factor for this finding is the threefold sample size of conventionally ventilated patients in the study by Dobyns and colleagues [14]. On the other hand, this analysis only concerned patients treated with HFO and CV without iNO.

In all studies, oxygenation significantly improved over 72 hours for patients treated with high-frequency oscillators [12,14,16]. A lack of uniform data on oxygenation, however, prevented analysis of pooled data. This finding is in contrast with that reported for preterm neonates. The systematic reviews and meta-analyses overall provide no evidence that HFO as the initial ventilation strategy offers important advantages over CV in terms of preventing chronic lung disease in preterm infants with acute pulmonary dysfunction [17-22].

The level of evidence proved moderate to good in three studies [12,14,15]. The study by Jaarsma and colleagues was stopped halfway through as both physicians and nurses preferred biphasic positive airway pressure [13]. This was designated level 1 evidence because of the high risk of bias. Likewise, the study by Carman and colleagues was designated level 1 evidence because the randomization failed for the demographic variable age [16].

The strengths of the present review include a comprehensive search strategy, broad inclusion criteria (resulting in a representative, heterogeneous population) and assessment of clinically important outcomes. In addition, we pooled the data. This statistical approach is also allowed for quasi-experimental, nonrandomized studies - such as the study by Dobyns and colleagues [14] - in which randomization of groups was not possible or failed [23]. Meta-analytic techniques in the analysis of nonrandomized studies have been criticized for their potential to perpetuate the individual biases of each study and to give a false impression of cohesion in the literature, thus discouraging further research [24]. The counter-argument is that statistical quantification and pooling of results from many studies helps to identify reasons for variability, inconsistency or heterogeneity in the literature, and thus may encourage further research [23,25]. Nevertheless, the pooled results of the present study should be interpreted cautiously in view of the diversity in patient groups, sample sizes, randomization methods, types of ventilators and ventilation strategies.

The reviewed RCTs cannot easily be compared owing to the heterogeneity in age, underlying disease and study outcomes. We would therefore recommend setting up studies investigating the best ventilation strategy for specific age categories or underlying pathology [1]. Furthermore, as mortality is rather low, longer-term outcome measures others than the short-term outcome measures studied in the present review should be considered, such as pulmonary function, neurocognitive development and cost-effectiveness. Internationally consensus on the most appropriate outcome measures should be reached.

## Conclusions

The available literature does not provide sufficient evidence on the best ventilation mode in critically ill children beyond the newborn period. High-frequency ventilation (HFO and VDR) provided better oxygenation after 72 hours than did CV. There is no evidence that high-frequency ventilation would reduce mortality and LOV.

## Key messages

• There is no evidence for the best ventilation mode in critically ill children beyond the newborn period up to 18 years.

• The different modes have not yet been investigated in (large) groups of children.

• Oxygenation significantly improved over 72 hours for patients treated with high-frequency oscillators.

• Longer-term outcome measures such as pulmonary function and neurocognitive development should be considered.

## Abbreviations

CI: confidence interval; CV: conventional ventilation; FiO_2_: fraction of inspired oxygen; HFO: high-frequency oscillation; iNO: inhaled nitric oxide; LOV: length of ventilation; OR: odds ratio; PC: pressure control; pCO_2_: partial arterial pressure of carbon dioxide; PF: PaO_2_/FiO_2 _ratio; pO_2_: partial pressure of oxygen; PS: pressure support; RCT: randomized controlled trial; SaO_2_: saturation of oxygen; VDR: volume diffusive respirator; VS: volume support; WMD: weighted mean difference.

## Competing interests

The authors declare that they have no competing interests.

## Authors' contributions

AD and DT conceived of and designed the study. AD and EI were involved in data acquisition, analysis, and interpretation and drafted the manuscript. DT and IR critically revised the manuscript for important intellectual content. All authors read and approved the final manuscript.

## Supplementary Material

Additional file 1 **Search strategy**. Word file containing the complete search strategy. Click here for file

Additional file 2 **Evaluation form of RCTs**. Word file containing a list of criteria for assessing the quality of RCTs. Click here for file
