# Supplementary material for: Invasive ventilation modes in children: a systematic review and meta-analysis
Source: Crit Care. 2011 Jan 17;15(1):R24. doi: 10.1186/cc9969 (PMC3222058; doi:10.1186/cc9969)
Supplement: Additional file 1 — Search strategy. Word file containing the complete search strategy. [file cc9969-S1.DOC]

**Additional file 1: Search strategy**

PubMed

(artificial respiration[mesh] OR artificial respir*[tw] OR mechanical ventil*[tw] OR high frequency respirat*[tw] OR liquid ventilat*[tw] OR pressure respirat*[tw] OR positive airway pressure*[tw] OR pressure breath*[tw] OR pressure ventilat*[tw] OR ventilator wean*[tw] OR ventilation wean*[tw] OR ventilators, mechanical[mesh] OR pulmonary ventilat*[tw] OR respirator*[tw] OR ventilator*[tw]) AND (instrument*[tw] OR device*[tw] OR mode[tw] OR modes[tw]) AND (infan*[tw] OR child*[tw] OR adolescen*[tw] NOT adult[mesh]) AND (randomized controlled trial*[tw] OR randomized controlled trial*[pt] OR rct[tw])

Hits: n=403

EMBASE

(artificial AND 'ventilation'/exp OR (artificial OR mechanical OR 'high frequency' OR 'liquid ventilation' OR 'liquid ventilator' OR pressure OR 'positive airway pressure' OR wean* OR pulmonary) NEAR/3 (breath* OR ventilat* OR respirat*) OR respirator*:de,ab,ti OR ventilator*:de,ab,ti) AND (instrument*:de,ab,ti OR device*:de,ab,ti OR mode:de,ab,ti OR modes:de,ab,ti) AND ([randomized controlled trial]/lim OR 'randomized controlled trial':ti,ab,de OR rct:ti,ab,de) AND ([newborn]/lim OR [infant]/lim OR [preschool]/lim OR [school]/lim OR [child]/lim OR [adolescent]/lim) NOT [adult]/lim NOT [aged]/lim AND [humans]/lim

Hits: n=250
