# Supplementary material for: Invasive ventilation modes in children: a systematic review and meta-analysis
Source: Crit Care. 2011 Jan 17;15(1):R24. doi: 10.1186/cc9969 (PMC3222058; doi:10.1186/cc9969)
Supplement: Additional file 2 — Evaluation form of RCTs. Word file containing a list of criteria for assessing the quality of RCTs. [file cc9969-S2.DOC]

**Additional file 2: Evaluation form of RCTs**

Assessment of the quality of a randomized controlled trial (RCT)

1. Was the intervention assignment truly random?
2. The person who includes subjects in the study should be unaware of the randomization order; was that the case?
3. Were patients blinded to treatment allocation?
4. Were caregivers blinded to treatment allocation?
5. Were persons measuring the outcomes blinded?
6. Were the groups comparable at baseline?
7. Was complete follow-up available for a sufficient proportion of all included patients? (loss-to-follow-up)
8. Were all included patients analyzed in the group to which they were randomized? Intention to treat analysis
9. Other than the intervention, was all care that patients received the same?

Intermediate conclusion

1. Are the results of the study valid and relevant?
2. Results. For each primary and secondary outcome, a summary of results for each group, and the estimated effect size and its precision (e.g. 95% CI)

Dichotomous outcomes

Continuous outcomes

Applicability in healthcare

1. Is the result found applicable to the situation in the Netherlands?
2. To which echelon(s) can the result be applied?
3. Conclusion
